# Profiling of three *H3F3A*-mutated and denosumab-treated giant cell tumors of bone points to diverging pathways during progression and malignant transformation

**DOI:** 10.1038/s41598-021-85319-x

**Published:** 2021-03-11

**Authors:** Marc Hasenfratz, Kevin Mellert, Ralf Marienfeld, Alexandra von Baer, Markus Schultheiss, P. D. Roitman, L. A. Aponte-Tinao, Burkhard Lehner, Peter Möller, Gunhild Mechtersheimer, Thomas F. E. Barth

**Affiliations:** 1 Institute of Pathology, University of Ulm, Albert-Einstein-Allee 11, 89081 Ulm, Germany; 2 Department of Trauma and Orthopaedic Surgery, University of Ulm, Ulm, Germany; 3 Pathology Department, Italian Hospital of Buenos Aires, Buenos Aires, Argentina; 4 Institute of Orthopaedics ‘‘Carlos E. Ottolenghi’’, Italian Hospital of Buenos Aires, Buenos Aires, Argentina; 5 Department of Orthopaedics and Trauma, University of Heidelberg, Heidelberg, Germany; 6 Institute of Pathology, University of Heidelberg, Heidelberg, Germany

**Keywords:** Cancer, Bone cancer, Cancer genetics, Cancer, Genetics research, Cancer, Bone cancer

## Abstract

Giant cell tumor of bone (GCTB) is a locally aggressive lesion of intermediate malignancy. Malignant transformation of GCTB is a rare event. In 2013, the humanized monoclonal antibody against receptor activator of nuclear factor-κb-Ligand (RANKL) denosumab was approved for treatment of advanced GCTB. Since then, several reports have questioned the role of denosumab during occasional malignant transformation of GCTB. We report on three patients with *H3F3A*-mutated GCTBs, treated with denosumab. The tissue samples were analysed by histomorphology, immunohistochemistry, and in two instances by next generation panel sequencing of samples before and after treatment. One patient had a mutation of *ARID2* in the recurrence of the GCTB under treatment with denosumab. One patient developed a pleomorphic sarcoma and one an osteoblastic osteosarcoma during treatment. Sequencing revealed a persisting *H3F3A* mutation in the osteosarcoma while the pleomorphic sarcoma lost the *H3F3A* mutation; however, a *FGFR1* mutation, both in the recurrence and in the pleomorphic sarcoma persisted. In addition, the pleomorphic sarcoma showed an *AKT2* and a *NRAS* mutation. These data are inconclusive concerning the role denosumab plays in the event of malignant progression/transformation of GCTB and point to diverging pathways of tumor progression of GCTB associated with this treatment.

## Introduction

Giant cell tumor of bone (GCTB) was first described by Jaffe et al.^1^ in 1940 and makes up about five percent of all primary bone lesions^2,3^; GCTB is typically located in the epiphyseal region of the long tubular bones such as the distal femur and the proximal tibia^3,4^. Based on the WHO-Classification for tumors of soft tissue and bone, GCTB is considered to be an aggressive and rarely metastasizing tumor and therefore is regarded as tumor of intermediate malignancy (ICD-O: 9250/1)^5^. Metastatic lesions most commonly occur in the lungs^4,6^. In rare cases, a high-grade malignant neoplasia is identified arising in GCTB (primary malignancy in GCTB or after previous radiotherapy (in both instances classified as ICD-O: 9250/3)).

Histologically, the GCTB mainly consists of three different cell types. These are the neoplastic spindle-shaped stromal cells that show enhanced synthesis of receptor activator of nuclear factor-κb ligand (RANKL) and the large multinucleated osteoclast-like giant cells and their monocytic precursors expressing the corresponding receptor, *i.e.,* receptor activator of nuclear factor-κb (RANK)^7,8^. The stroma cells are the neoplastic component of GCTB and harbour a characteristic point mutation at the histone gene *H3F3A* leading to a substitution of glycine by tryptophan at position 34 (G34W). The increased synthesis of the RANKL by the neoplastic cells leads to enhanced bone resorption by the osteoclast-like giant cells through the RANK/RANKL signalling pathway, which in turn are responsible for the locally aggressive growth.

Intralesional curettage is the primary treatment option for GCTB^6^. The rate of local recurrence after intralesional curettage ranges from 30 to 40%^3,9^. Denosumab was first approved for the treatment of osteoporosis under the brand name PROLIA in 2010 and was later approved for the treatment of GCTB in 2013 under the brand name XGEVA. Denosumab is a humanized monoclonal IgG2-anti-RANK-Ligand-antibody. This monoclonal antibody binds to RANKL and inhibits the interaction between the spindle-like stromal cells and the osteoclast-like giant cells, and thereby prevents local bone resorption, thus mimicking the effect of osteoprotegerin, a physiological RANKL-antagonist^6,7^.

## Clinical case presentation

Patient one is a female, first diagnosed with GCTB in 2015 at the age of 33. The tumor measured 12 cm and was located in the pelvis; she was treated by a complete (R0) resection. In 08/2016 a denosumab treatment was started (120 mg subcutaneously every 4th week) until 06/2017 due to recurrence in the pelvis confirmed by a biopsy. The patient is well and shows no further signs of progression.

Patient two is a male that was diagnosed with a tumor of the sacrum measuring 13 cm in 03/2014 at the age of 20. A biopsy was performed and the diagnosis of GCTB was confirmed by detection of the *H3F3A G34W* mutation. In 04/2014 an incomplete resection of the tumor with instillation of alcohol 90% was performed. A fistula, which developed shortly after the first surgery, was resected.

In 12/2014, nine months after resection of the GCTB, a recurrence was diagnosed and denosumab treatment was started with 120 mg every 4 weeks until 05/2016. In 2017 the patient underwent palliative surgery. A CT-Scan showed a large local recurrence and pulmonary as well as liver lesions highly suspicious of metastases. The tumor mass was resected in 07/2017 and a high-grade osteosarcoma with angioinvasion harbouring the *H3F3A G34W* mutation was diagnosed.

The patient died in 08/2017 due to tumor progression and sepsis.

Patient three, a woman, is a follow-up initially published by Aponte-Tinao et al.^10^ in 2015. These authors reported the case of a 20-year-old female who was first diagnosed with GCTB in 2009. The GCTB was located at the right proximal tibia and was treated by intralesional curettage. She was diagnosed with a recurrence of GCTB about one year later in 2010 and was treated by *en-bloc* resection. The following two years were uneventful until a follow-up CT-scan showed a second recurrence in 2013 and denosumab therapy was started. The first application was a subcutaneous dose of 360 mg followed by 120 mg subcutaneously every 4 weeks. About one year after beginning of treatment with denosumab, the patient noticed a palpable, painful mass in the popliteal fossa. A CT-Scan showed that the mass included two sections of different density. An open biopsy was performed and histologic workup showed a high-grade undifferentiated pleomorphic sarcoma besides the GCTB. Subsequently an above-knee amputation was performed. Several tissue blocks were available for further histological analysis of the resection specimen. The patient is well and shows no signs of progression.

## Methods

The samples were analysed by conventional histology using haematoxylin–eosin (HE) staining of sections of paraffin-embedded tissue. Immunohistochemistry was performed as described using a mutation specific monoclonal antibody for detection of the *H3F3A G34W* mutation (Anti H3.3 G34W clone 31-1145-00; RevMab Biosciences, San Francisco, CA, USA; dilution 1:400); for detection of proliferation indices the Ki-67 antibody (M7240, Dako, Glostrup, Denmark; dilution 1:200) was applied^11^.

The alkaline phosphatase/RED detection system (Dako) was used for immunohistochemistry on formalin-fixed and paraffin-embedded tissue via the avidin–biotin-complex-method. The samples were pseudonymized according to the German law for correct usage of archival tissue for clinical research. The research was approved by the local ethics committee of the University of Ulm (reference 369/17) and was in compliance with the ethical principles of the World Medical Association Declaration of Helsinki. Informed consent was obtained from all patients.

### Isolation of tumor DNA from FFPE tissue

For isolation of genomic DNA from the formalin fixed paraffin embedded (FFPE) tissue samples, 5 μm tissue slices were transferred to glass slides. To estimate the area containing the tumor, HE stained FFPE tissue slices (2 µm) were validated by an expert pathologist. The tumor-harbouring areas of the FFPE tissue were subjected to a DNA extraction procedure using the QIAamp DNA FFPE tissue kit (QIAGEN, Hilden, Germany) according to manufacturer’s instruction. DNA purity and concentration were determined fluorometrically (Qubit 2.0; Invitrogen, Carlsbad, CA, USA).

We further performed Sanger-Sequencing for H3F3A G34W for all tissue samples of patient 1 and patient 3. Gene sequencing (Sanger) was performed according to a diagnostic standard protocol. The cell line A498 was used as negative control. The graphs were generated with FinchTV 1.4.0 (Geospiza Inc., Seattle Washington, USA)^12^.

### Next generation sequencing

For molecular characterization of both tumor tissue and ctDNA, we employed a targeted re-sequencing methodology using the GeneRead V2 chemistry (QIAGEN, Hilden, Germany) and a custom-made re-sequencing panel including primers for all exons of *a panel of* 37 genes (primer sequences and locations of target areas are available upon request). Target enrichment, amplicon processing, and library generation were performed according to the manufacturer’s instructions. For target enrichment, we included 10–40 ng (DNA from FFPE tumor and non-neoplastic tissue). Successful target enrichment and library generation was checked using the High Sensitivity DNA kit on a bioanalyzer device (Agilent, Santa Clara, CA, USA). Libraries were diluted to 10 pM solutions and the sequencing was performed on a MiSeq platform (Illumina, San Diego, CA, USA) using the V2 chemistry. Mean read depth on target region was 2000–8000-fold and 99% of bases were covered at 96–100% on average. The resulting fastq files were subjected to further analysis using the GeneRead web based analysis tool (http://ngsdataanalysis.sabiosciences.com/NGS2/), the Biomedical Workbench software package (QIAGEN, Hilden, Germany), and the Variant Studio software (Illumina, San Diego, CA, USA)^13^. All identified mutations were manually re-analyzed using the Integrated Genome Viewer Software (Broad Institute, MA, USA).

Single nucleotide polymorphisms (SNP) detected in non-tumor-tissue were excluded from further analysis.

## Results

### Patient 1

#### Morphology

**Figure 1:**
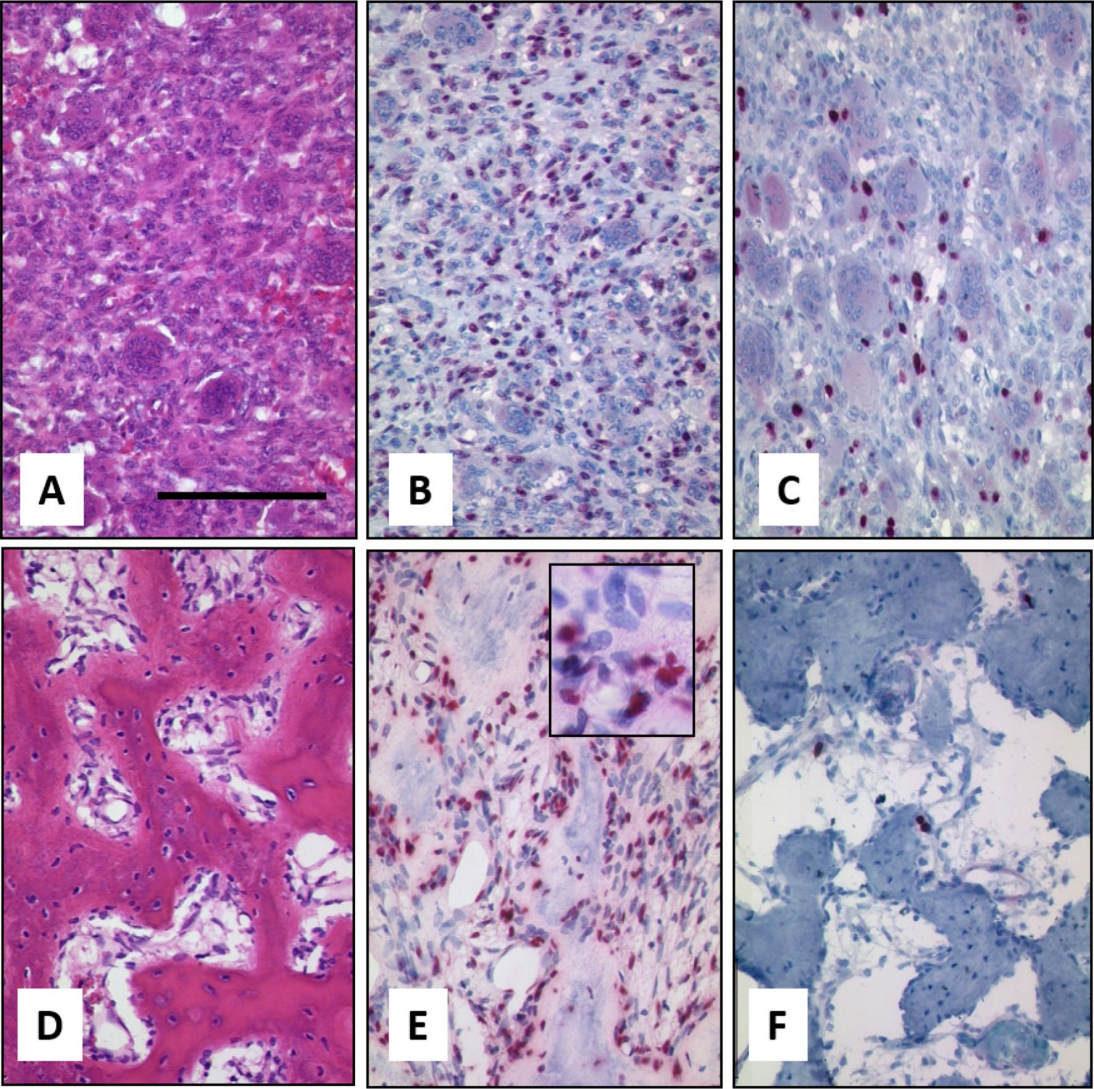
Morphology of Case 1. **(A)** Typical GCTB with osteoclastic giant cells and intermingled mononuclear cells before therapy (bar = 100 µm). Neoplastic mononuclear cells are positive in a H3F3A G34W staining in the nuclei **(B)** and about 5% of cells are Ki-67 positive **(C)**. After denosumab therapy GCTB shows induction of neoformated osteoid and complete reduction of giant cells **(D)**, while H3F3A G34W-positive cells are still present **(E)**; insert shows H3F3A-negative spindle cells. Proliferation has dropped to 1% in a Ki67 staining **(F)**.

Tissue blocks of the primary biopsy, the resection specimen, and the recurrence under denosumab therapy were available. Histological analysis of the biopsy and resection specimen showed a giant cell tumor with typical morphology, consisting of osteoclastic giant cells and a mononuclear spindle cell compartment. The primary tumor showed a strong positivity for the *H3.3 G34W* detecting antibody in the nuclei of the stromal compartment. Nuclei of osteoclastic giant cells were negative. A second mononuclear cell population was detected being negative for H3.3 G34W which most probably presents osteoclastic precursors. Ki-67 rate was about 5% in the mononuclear cell population. After treatment the tumor revealed a dramatic change in morphology. The number of H3.3 G34W-positive osteoblastic cells was greatly reduced; positive mononuclear cells were still detectable along strands of neoformated osteoid. We detected an intermingled spindle cell compartment negative for H3.3 G34W. The Ki-67 index dropped to less than 1% (Fig. 1). The presence of the *H3F3A G34W* mutation was further proven by Sanger sequencing.

Panel sequencing of microdissected tissue of the resected tumor and the biopsy of the recurrence revealed a mutation of *ARID2* (chromosome 12; position (GRCh37) 46205208, c.292G > A; p.E98K) with an allele frequency of 48% in the tumor recurrence under denosumab treatment. Neither the non-tumorous tissue nor the GCTB before treatment with denosumab harboured this mutation.

### Patient 2

#### Morphology

The biopsy and resection specimen revealed a GCTB with typical morphology of osteoclastic giant cells and mononuclear spindle cells. Ki-67 rate was about 5%. Immunochemistry and sequencing confirmed the diagnosis of GCTB with positive staining for the mutation *H3F3A G34W*. This diagnosis was verified by Sanger-Sequencing.

**Figure 2:**
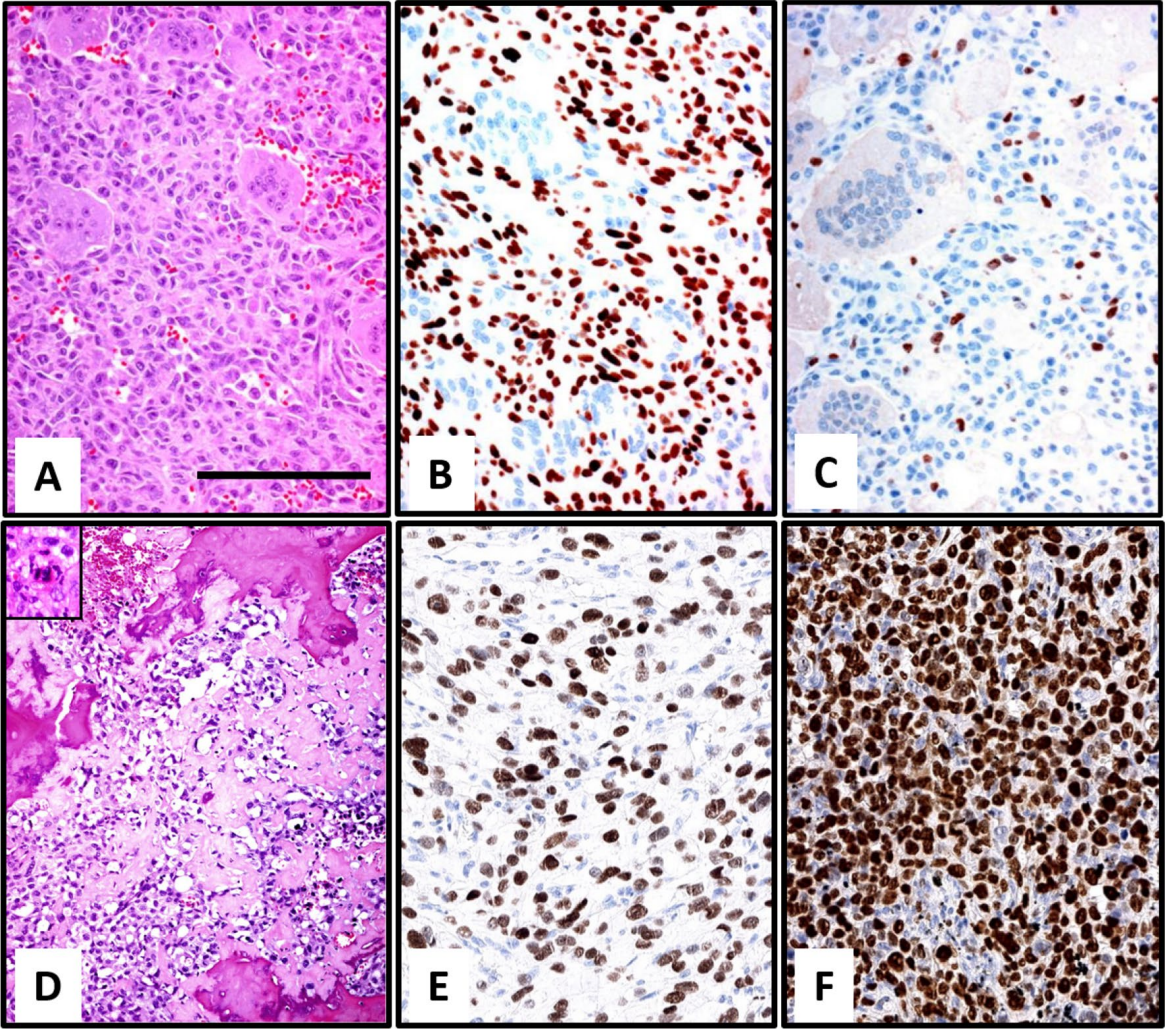
Morphology of Case 2. Typical morphology of GCTB with osteoclastic giant cells and mononuclear spindle cells **(A)** neoplastic mononuclear cells with positive H3F3A G34W staining **(B)** (bar = 100 µm). Staining for Ki-67 shows about 5% positive cells **(C)**. The sarcoma showed presents with polymorphic tumor cells andatypical mitotic figures with necrosis intermingled lace-like neoplastic osteoid intermingled with necrotic bone. An atypical mitotic figure is shown in the insert **(D)**. The sarcoma harboured the *H3F3A G34W* mutation **(E)** and had a high rate of Ki-67 **(F)**.

Morphology of the resected recurrence revealed a pleomorphic high-grade sarcoma with necrosis and presence of atypical mitotic figures. The sarcoma showed intermingled lace-like neoplastic osteoid and was classified as a high-grade osteosarcoma; an angioinvasion was present. The sarcoma harboured the *H3F3A G34W* mutation as shown by immunohistochemistry in all tumor cells and a Ki-67 index of 90% (Fig. 2). This presence of the mutation was further confirmed by Sanger sequencing for *H3F3A G34W* mutation.

### Patient 3

#### Morphology

The morphology of this tumor has been described in details by Aponte et al.^10^. In brief, the initial GCTB revealed a typical morphology with osteoclastic giant cells and a mononuclear *H3F3A G34W-*positive neoplastic tumor population as shown by immunohistological staining and sequencing both of the primary tumor and the recurrence. The Ki-67 index was about 5%. The sarcoma after denosumab treatment showed a high-grade sarcoma (not otherwise specified) with spindle-like to pleomorphic tumor cells and extensive necrosis and a Ki-67 index of 50%.

The sarcoma was negative in an immunohistochemical staining with the monoclonal antibody for detection of H3F3A G34W.

**Figure 3:**
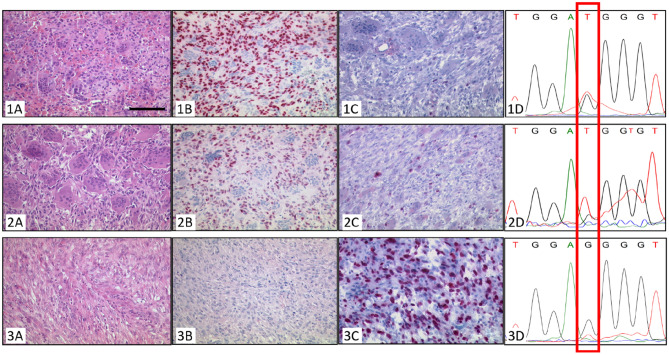
Morphology of Case 3. (1) Biopsy. (2) Resection specimen. (3) Sarcoma. Typical GCTB with osteoclastic giant cells and intermingled mononuclear cells in the biopsy **(1A)** and the resection specimen **(2A)** as well as neoplastic mononuclear cells with positive H3F3A G34W staining in the nuclei **(1B,2B)** (bar = 100 µm). Staining for Ki-67 shows about 1% of positive cells **(1C,2C)**. Sanger-Sequencing for H3F3A G34W shows the mutation specific thymine in tissue of the biopsy **(1D)** as well as the resection specimen **(2D)**. The sarcoma showed spindle to pleomorphic tumor cells and extensive necrosis **(3A)** and a high Ki-67 index **(3C)**, however it did not stain positive for H3F3A G34W **(3B)**. Sanger-sequencing of DNA from microdissected tissue of the H3F3A G34W-negative tumor showed a very weak peak for a mutation-specific thymine **(3D)**.

Sequencing of DNA from microdissected tissue of the H3F3A G34W-negative tumor showed a very weak peak for a mutation-specific thymine that was below the detection threshold as shown in Fig. 3.

**Figure 4:**
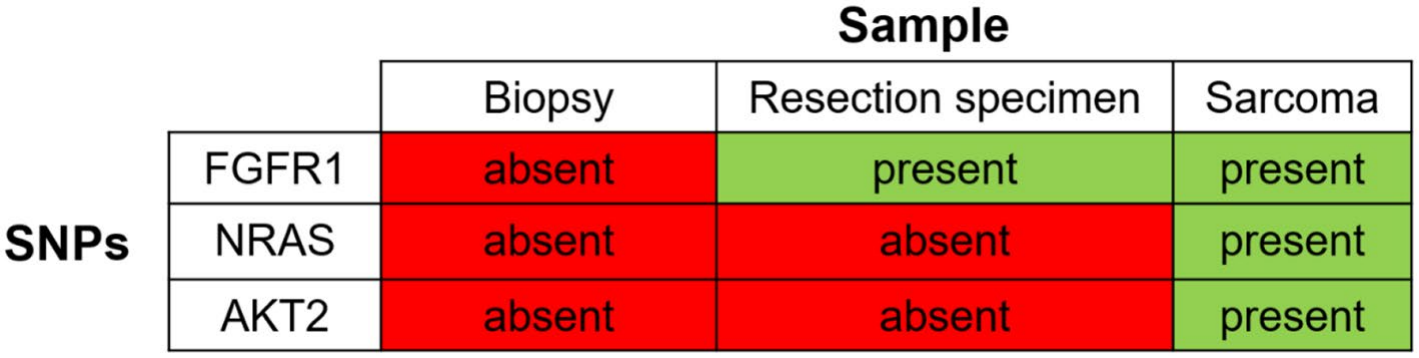
Summary of SNPs found in the different tissue samples (Case 3). SNPs in the resection: FGFR1 (chromosome 8; position (GRCh37) 38287303; c354G > A; p.E118). SNPs in the pleomorphic sarcoma: FGFR1 (chromosome 8; position (GRCh37) 38287303; c354G > A; p.E118); AKT2 (chromosome 19; position (GRCh37) 40742052); NRAS (chromosome 1; position (GRCh37) 115256669).

In the panel-sequencing we found a mutation of *FGFR1* (chromosome 8; position (GRCh37) 38287303; c354G > A; p.E118) in the resection specimen of the GCTB and in the sarcoma with an allele frequency of 10%, which was not found in the biopsy. We found two additional mutations in the sarcoma tissue for *AKT2* (chromosome 19; position (GRCh37) 40742052) and *NRAS* (chromosome 1; position (GRCh37) 115256669) with a frequency of 26% and 48%, respectively; these two mutations were not present in the initial biopsy or in the resection specimen (Fig. 4) and present intronic mutations with no effect on the protein structure. Supplementary Figs. 1 and 2 summarize the clinical and molecular findings.

## Discussion

GCTB is characterized by an enhanced paraneoplastic secretion of RANKL, leading to a shift of normal bone homeostasis to enforced bone resorption due to an increased number of induced non-neoplastic osteoclasts^3,14^. Denosumab was first approved for treatment of GCTB in 2013 in advanced stage and is a humanized monoclonal IgG2 antibody. Through specific binding to RANKL denosumab blocks the RANK/RANKL signalling. This treatment leads to striking changes in morphology of GCTB characterized by reduction of both, the neoplastic cell compartment and the osteoclasts^14^. Due to blockage of the RANK/RANKL axis enhanced osteoid neoformation of bone is observed by *H3F3A*-non-mutated osteoblasts and fibroblastoid spindle cells^11^.. Mak et al. showed that denosumab reduced the number of osteoclasts and RANKL secretion in vitro, however the neoplastic stromal cells continued to grow after reduction of denosumab, even though at a slower proliferation rate^15^. The authors conclude that after denosumab treatment the *H3F3A*-mutated tumor cell pool may reside in the tissue and be reactivated during recurrence^15^. Besides the role of the RANK axis in the control of bone remodelling and as a crucial factor in GCTB growth, RANKL has an important role for the differentiation of B and T cells as well as for the survival of dendritic cells^16,17^; by this mechanism, enhanced RANKL synthesis in GCTB may lead to immunosuppression and therefore promote neoplastic transformation^18^.

On the other hand, nuclear factor κb upregulation by enhanced RANK signalling is discussed to interfere with regulation of oncogenes^18^. It has been shown that RANKL upregulates the expression of semaphorin 3A^20^, and may thereby affect growth of cartilage and bone^21^. Furthermore, in a comparative proteome study of GCTB before and after denosumab therapy several differentially expressed proteins were identified including metalloproteinase 9 being downregulated after denosumab treatment^19^. Interestingly, we noted a reduction of proliferation of the GCTB after treatment in the recurrences of patient one, however we further detected a *H3F3A-*negative mononuclear population most probably corresponding to re-populating osteoblasts. The suppression of RANK signalling may therefore generate a micro milieu favourable for malignant transformation.

In osteoporosis, denosumab treatment leads to significant increase in bone density; up to now no cases have been described of malignant tumors in patients treated with denosumab for osteoporosis. GCTB and osteoporosis are treated with different doses of denosumab. In osteoporosis 60 mg of denosumab is administered in a subcutaneous injection every six months. For treatment of GCTB a loading dose of 360 mg may be administered, followed by a subcutaneous injection of 120 mg every 4 weeks. The prescribed dose for GCTB is therefore estimated 12-times higher than the dosage used for treatment of osteoporosis. Since the approval for treatment of GCTB there have been at least 15 described cases of malignant transformation of GCTB in association with denosumab treatment^10,20–26^. Due to these reports the question arises whether the sarcoma is clonally linked to the primary tumor. In patient one we detected an *ARID2* mutation limited to the recurrence of the GCTB after denosumab treatment. This finding suggests that mutated *ARID2* is a marker during tumor progression under denosumab treatment in this *H3F3A*-mutated GCTB, although no sarcomatous transformation is present. In line with this finding is that *ARID2* was identified to play a critical role in the differentiation of osteoblasts and that a mutation may interfere in this process^27^. The second patient reported was characterized by a persisting *H3F3A* mutation in the relapse as well as in the osteosarcoma arising in the sacrum. This finding points to a clonal evolution of the sarcoma under denosumab treatment; the *H3F3A* mutation can be regarded as a clonal marker in this case. In rare cases, *H3F3A* mutations have been described in osteosarcoma of the epiphyseal region of mostly older patients as shown by Koelsche et al.^28^. This finding is in line with a possible transformation of a pre-existing GCTB completely overrun by the sarcoma. In contrast, in patient three, a different mutational profile during progression emerged regarding the *H3F3A* mutation. The initial GCTB in the biopsy and in the recurrence was *H3F3A*-mutated, while the sarcoma was negative for this mutation as shown by sequencing and immunohistochemical staining. However, the recurrence of the GCTB and the sarcoma revealed an overlapping mutation for *FGFR1*, which was not present in the first biopsy. In addition, we detected a mutation in *AKT2* and *NRAS* in the sarcoma. This may point to the evolution of a high-grade sarcoma from a *H3F3A*-negative, but *FGFR1*-positive subclone with acquisition of additional mutations in *AKT2* and *NRAS* during tumor progression. One explanation is a transformation of the *H3F3A*-negative mononuclear cells residing in the tumor after denosumab treatment. Although the detected mutations in *NRAS* and *AKT2* are intronic in the presented GCTB with no effect on the cDNA level these two genes have been shown to play a role in bone physiology and osteosarcomas. While *NRAS* mutations have been described in dysplastic bone formation^29^, *AKT2* has been shown to play a role in tumor growth by inhibiting cisplatin-induced apoptosis in primary osteosarcomas^30,31^. Furthermore, Zhu et al. described an enhanced expression of *AKT2* in osteosarcoma and that this finding is associated with a more aggressive clinical behaviour and worse outcome^32^.

Based on our analysis we cannot determine whether or not the transformation is a spontaneous event and occasionally associated with denosumab treatment. Large studies with a total of 2315 patients with GCTB have shown a cumulative incidence of primary malignant giant cell tumor of Bone (PMGCTB) of 1.6% in GCTB compared with 2.4% for secondary malignant giant cell tumor of bone (SMGCTB) following radiotherapy^33^. PMGCTB usually occurs next to an area of benign GCTB whereas SMGCTB occurs superimposed on a previously treated benign GCTB^34^. Several studies showed that patients with PMGCTB were of older age compared to patients with SMGCTB^34,35^. The clinical presentation of PMGCTB and SMGCTB is comparable^36^. Radiologically PMGCTB is often not distinguishable from benign GCTB whereas SMGCTB often presents findings suspicious of malignancy^35^.

In a further study on 532 patients receiving denosumab the number of patients with sarcomatous transformation was limited to four patients (1%)^26^.

Scotto di Carlo et al. reported in a recent study that GCTB shows a more malignant phenotype in a patient with Paget disease of bone which further points to malignant behaviour of GCTB depending on further co-factors^37^.

Whether the patients reported in literature with sarcomas after denosumab treatment fall in the category of being spontaneously transformed or being associated with denosumab therapy is yet not clear since 12 of them did not receive additional radiotherapy.

In conclusion, our findings point to different molecular profiles of GCTB associated with denosumab treatment and raise the question whether these events are spontaneous or associated with denosumab treatment. To clarify this question, more studies should be performed of GCTB in progression with and without denosumab treatment excluding those patients who received radiotherapy.

## Supplementary Information

Supplementary Information.

## Data Availability

The data that support the findings of this study are available from the corresponding author upon reasonable request.
