# Supplementary material for: Profiling of three H3F3A-mutated and denosumab-treated giant cell tumors of bone points to diverging pathways during progression and malignant transformation
Source: Sci Rep. 2021 Mar 11;11:5709. doi: 10.1038/s41598-021-85319-x (PMC7952552; doi:10.1038/s41598-021-85319-x)
Supplement: Supplementary file 1 — Supplementary Information. [file 41598_2021_85319_MOESM1_ESM.docx]

**Profiling of Three *H3F3A*-Mutated and Denosumab-Treated Giant Cell Tumors of Bone Points to Diverging Pathways During Progression and Malignant Transformation**

Marc Hasenfratz^1^, Kevin Mellert^1^, Ralf Marienfeld^1^, Alexandra von Baer^2^, Markus Schultheiss^2^, P. D. Roitman^3^, L. A. Aponte-Tinao^4^, Burkhard Lehner^5^, Peter Möller^1^, Gunhild Mechtersheimer^6^ and Thomas F. E. Barth^1*^

| CASE | Sex | Age at first diagnosis | Location | Duration of denosumab treatment | Mutations found after denosumab treatment |
| --- | --- | --- | --- | --- | --- |
| 1 | female | 33 | Pelvis | 11 months | *H3F3A, ARID2* |
| 2 | male | 20 | Sacrum | 18 months | *H3F3A* in the primary GCTB and the sarcoma |
| 3 | female | 15 | right Tibia | 13 months | *FGFR1, AKT2, NRAS* |

***Supplementary Figure 1:*** Summarized clinical and molecular findings


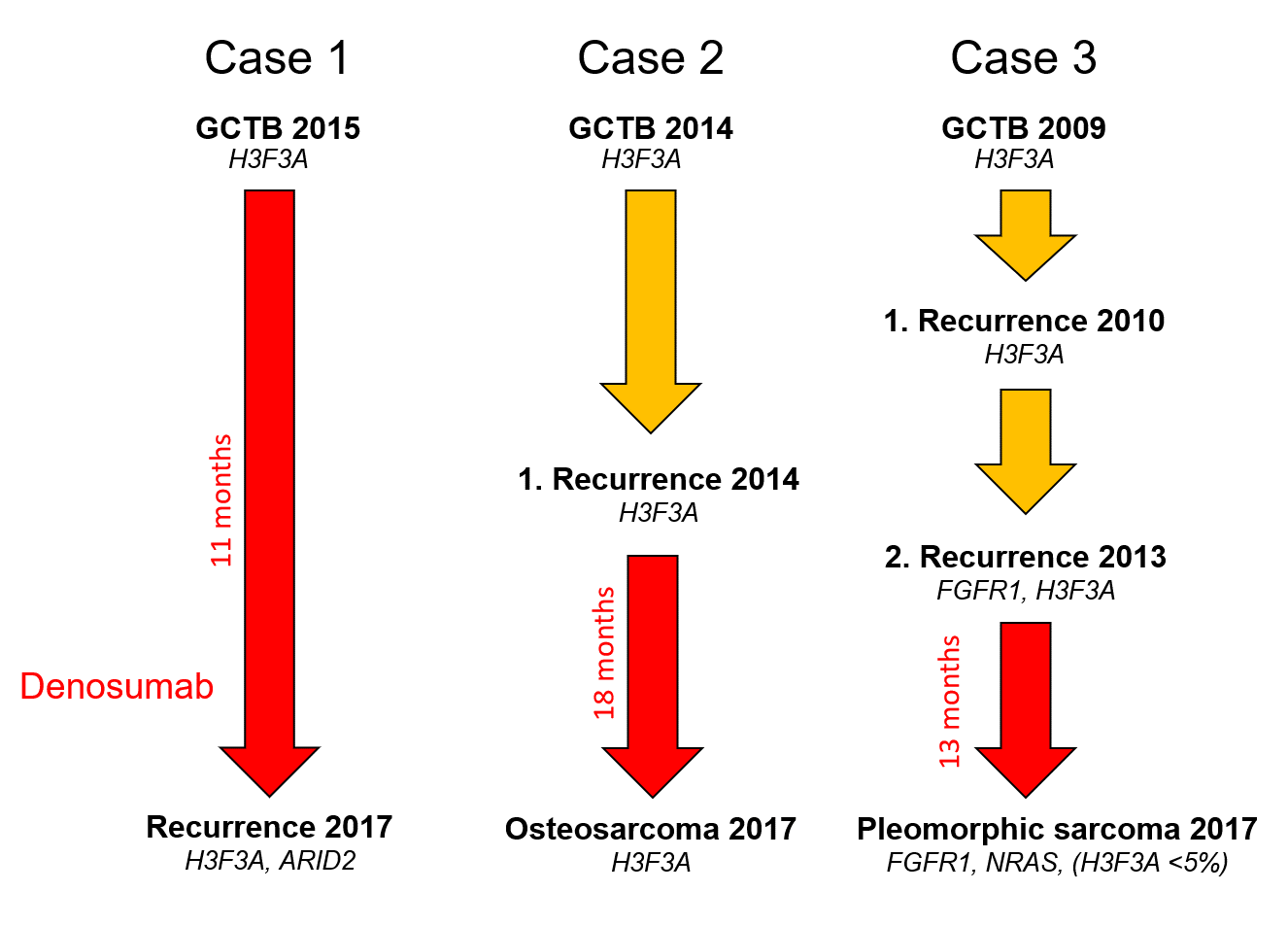


***Supplementary Figure 2:*** Timeline showing chronical order of the disease of the three patients.

| AKT2 | FGFR3 | NF1 |
| --- | --- | --- |
| ARID2 | GNA11 | NF2 |
| BRAF | GNAQ | NRAS |
| CASP8 | HRAS | PDGFRA |
| CDK4 | IDH1 | PIK3CA |
| CDKN2A | IDH2 | PIK3R1 |
| CTNNB1 | KIT | PTEN |
| EGFR | KRAS | PTPN11 |
| ERBB2 | MAP2K1 | RAC1 |
| ERBB3 | MAP2K2 | RB1 |
| ERBB4 | MDM2 | TP53 |
| FGFR1 | MET |  |
| FGFR2 | MITF |  |

***Supplementary information:*** 37 genes tested for SNPs by next generation panel sequencing on the custom panel from Illumina.
